# Exploring the Mode of Action of Bioactive Compounds by Microfluidic Transcriptional Profiling in Mycobacteria

**DOI:** 10.1371/journal.pone.0069191

**Published:** 2013-07-31

**Authors:** Paul Murima, Paola Florez de Sessions, Vivian Lim, Ahmad Nazri Mohamed Naim, Pablo Bifani, Helena I. M. Boshoff, Vasan K. Sambandamurthy, Thomas Dick, Martin L. Hibberd, Mark Schreiber, Srinivasa P. S. Rao

**Affiliations:** 1 Novartis Institute for Tropical Diseases, Singapore, Singapore; 2 Genome Institute of Singapore, A*STAR, Singapore, Singapore; 3 Tuberculosis Research Section, Laboratory of Clinical Infectious Diseases, National Institute of Allergy and Infectious Diseases, National Institutes of Health, Bethesda, Maryland, United States; 4 Department of Microbiology, National University of Singapore, Singapore, Singapore; 5 Novartis Institutes For Biomedical Research, Cambridge, Massachusetts, United States of America; Institut de Pharmacologie et de Biologie Structurale, France

## Abstract

Most candidate anti-bacterials are identified on the basis of their whole cell anti-bacterial activity. A critical bottleneck in the early discovery of novel anti-bacterials is tracking the structure activity relationship (SAR) of the novel compounds synthesized during the hit to lead and lead optimization stage. It is often very difficult for medicinal chemists to visualize if the novel compounds synthesized for understanding SAR of a particular scaffold have similar molecular mechanism of action (MoA) as that of the initial hit. The elucidation of the molecular MoA of bioactive inhibitors is critical. Here, a new strategy and routine assay for MoA de-convolution, using a microfluidic platform for transcriptional profiling of bacterial response to inhibitors with whole cell activity has been presented. First a reference transcriptome compendium of Mycobacterial response to various clinical and investigational drugs was built. Using feature reduction, it was demonstrated that subsets of biomarker genes representative of the whole genome are sufficient for MoA classification and deconvolution in a medium-throughput microfluidic format ultimately leading to a cost effective and rapid tool for routine antibacterial drug-discovery programs.

## Introduction

Since the early 20^th^ century, bioactive inhibitors used for anti-infective chemotherapy have been identified by phenotypic screens and further examined in complex biological systems [1]. Advances in genome sequencing, molecular biology and biochemistry led to an evolution from the traditional phenotypic screens to a more ‘reductionist’ target-based approach, which was thought to be more rational and efficient [2]. Despite the rapid identification of diverse, novel drug targets characterized by genetic tools [3], target-based anti-bacterial lead discovery has been less successful [4]–[6]. In many cases, these target-based screens reveal small molecules with potent activity against the purified target *in vitro* but fail to render anti-bacterial activity in both *in vitro* and *in vivo* models [4], [7].

The large-scale failure of genomics driven anti-bacterial lead discovery programs has led to the renaissance of empirical phenotypic screens for the identification of new chemotypes [6], [8], [9]. In contrast to target-based screening, molecules identified using this approach have the advantage of not only possessing desirable physicochemical properties from the beginning (such as cell penetration), but are also active against the relevant target in its intracellular context, under physiological conditions. Despite this key advantage, success in defining the target, mechanism of action (MoA), and the final lead optimization of hits derived from phenotypic screens has been low [4], [6].

One of the daunting tasks for medicinal chemists during hit to lead and lead optimization of hits, and scaffolds derived from whole cell screen, is to make sure that the compounds they are synthesizing also have similar MoA as that of the parent molecule. In order to understand the structure activity and property relationship (SAR and SPR) medicinal chemists synthesize multiple compounds in and around the parent molecule. It is very critical that the new molecules are acting in a similar way as that of the parent in order to get desired final effect. Currently, lead optimization of hits from phenotypic screens can only be best done with a known target. Although various approaches for MoA and target deconvolution have been established, including characterization of resistant mutants, biochemical affinity-based methods, genetic complementation, protein and DNA microarrays [10], target identification is still a challenging and inefficient task to support the early discovery process [6].

Until the last decade, MoA deconvolution was largely limited to model organisms whose metabolic pathways have been well characterized. Transcriptional profiling by microarray analysis has been used to analyze the MoA of early anti-bacterial [11], [12], anti-fungal [13], and anti-malarial compounds [14]. Despite the elegance of this approach for MoA deconvolution, it is not practical for use as a routine assay [15]–[17]. To benefit from the transcriptional profiling body of evidence we have established a miniaturized gene expression assay for efficient MoA deconvolution and discovery chemistry based on microfluidics. The microfluidic integrated fluidic circuits (IFC) contain tens of thousands of microfluidic-controlled valves and interconnected channels for transporting and combining cDNA molecules and qPCR reagents in complex patterns [18]. As a result of the miniaturization inherent in this approach, a single assay is capable of increasing the throughput of traditional qPCR by 2 orders magnitude using nanolitre reaction volumes compared to the standard techniques [19]. Here we report the application of this tool as a routine assay for MoA deconvolution, and its help in hit to lead and lead optimization of novel compounds obtained by phenotypic screens. We demonstrate that a minimal number of differentially expressed genes are sufficient to classify the MoA of novel chemical entities (NCE).

## Materials and Methods

### Bacterial cultures and RNA extraction

*Mycobacterium tuberculosis* (ATCC 27294) and *M. bovis* BCG (Pasteur) were grown with aeration at 37°C in Middlebrook 7H9 (Difco) liquid culture medium supplemented with 0.5% (w/v) bovine serum albumin fraction V, 0.2% dextrose, 0.08% sodium chloride, 0.5% glycerol, and 0.05%. tween 80 to mid-log phase. The mid log phase culture was concentrated and re-suspended to A_600_ nm of 0.3 (Amersham Ultrospec 3300). Antimicrobial compounds were added at either 0.5×, 1×, or 5× minimum inhibitory concentration (MIC) determined using a turbidimetric microplate assay. For microarray experiments *M. tuberculosis* H37Rv strain was anti-tubercular compounds were treated at different concentrations for 6 hours prior to isolation of RNA for analysis. All the details are provided in GEO accession # GSE46212. For real time qPCR experiments, *M. bovis* BCG cells were treated at 0.5× MIC_50_ of various anti-tubercular agents for 3 hours prior to isolation of RNA. For microfluidics experiments, *M. bovis* BCG cells were treated at 5× MIC_50_ of various anti-tubercular agents for 3 hours prior to isolation of RNA. Dimethyl sulfoxide (DMSO, drug vehicle) treated mycobacterial cultures were used as untreated controls in all experiments. The culture of cells treated/untreated with the inhibitor were pelleted in RNAlater (Qiagen) and bacterial RNA were extracted using the GTC/Trizol® method [20].

### Quantitative reverse transcription qPCR assay

The expression of genes selected as probes to represent the whole genome was quantified using real time qPCR, after normalizing the RNA expression levels to SigA as previously described [11].

### Microarray gene expression

Microarray analysis was performed as described [11]. The microarray data obtained for the compounds has been submitted to NCBI (GEO accession # GSE46212 and GPL1343). Expression ratios were calculated as the feature pixel median minus background pixel median for one color channel divided by the same for the other channel. In cases where more than 10% of the feature pixels were saturated, the feature pixel mean was used instead of the median. When the feature pixel mean did not exceed the background pixel mean by more than two standard deviation values (calculated from the background pixel distribution), the feature pixel median was used in the ratio without background subtraction. In cases where both color channels were near background (same criterion), the ratio value was set to “missing.” Expression ratios were transformed to the log base 2 for all further calculations.

### Microfluidic gene expression analysis

Using the previously described BioMark Fluidigm 96.96 microfluidic chip (Fluidigm, South San Francisco, Calif., USA) [19], we analyzed bacterial transcriptional response in the antibiotic or vehicle treated cells. Total RNA was isolated using Trizol and RNA was quantified using a Nanodrop and adjusted to 150 ng for reverse transcription using High-Capacity RT Kit (Applied Biosystems, 4368813), as per the manufacture's recommendations. Pre-amplification of the cDNA was performed using a Taqman PreAmp Master Mix Kit (Applied Biosystems, 4391128) as per the manufacture's recommendations, and the preamplified product was diluted at 1∶20. The pre-amplified product was used for the BioMark 96.96 Real-time PCR assay (Fluidigm) according to the manufacture's recommendations using Eva green DNA binding dye (Biotium). Fluidigm uses integrated fluidic circuits to generate a medium throughput for real-time PCR. In the BioMark platform 96 primer sets and 96 samples can be loaded and via the on-chip network of microfluidic channels, chambers and valves individual PCR reactions will be assembled simultaneously (a maximum of 9,216 reactions can be evaluated at one time). The complete mechanism of how microfluidic circuit and valve system technology work have been well described elsewhere (http://www.fluidigm.com/technology.html).

### Data processing and analysis

To identify differentially regulated genes, a measure of significance t-test was applied to the normalized data set. To discriminate genes that significantly deviated from the 1∶1 ratio (treated:vehicle), a minimum p-value of 0.05 was used. Differentially expressed genes were subsequently subjected to Benjamini and Hochberg correction to account for multiple experimental testing.

### Feature reduction

Feature reduction is the process of reducing the number of variables (in this case genes) defining a condition. Feature reduction often attempts to identify a minimum set of non-redundant features that are useful for classification. Microarray data of mycobacteria treated with a variety of antibacterial compounds were hierarchically clustered using Euclidian distance as a distance matrix and a minimal variance method (Ward linkage) to join adjacent clusters [21]. It can be summarized by the following five steps: a) Distances between all gene pairs are calculated, using Euclidean distance, b) The resulting distance matrix is thoroughly inspected to find the smallest distance between expression profiles, c) The corresponding genes are joined together in the tree and form a new cluster, d) The distances between the newly formed cluster and the other genes are recalculated, e) Steps b, c and d are iteratively repeated until all genes and clusters are linked in a final tree.

A hierarchical cut off was selected that gave 90 gene clusters. A single biomarker gene to represent each cluster was identified by finding the gene in the cluster with the best Pearson correlation against the cluster mean. In total, 90 genes were selected optimizing the number of biomarkers measured in the 96 probe fluidigm format while allowing for up to 6 control probes. The reduction method included following steps; a) computation of the mean expression of genes in a cluster in all conditions, b) computation of Pearson product moment correlation co-efficient to the cluster mean for each gene, and c) the selection of gene with the best correlation co-efficient in each cluster as the feature to represent the cluster.

The robustness of experimental set up was evaluated using Pearson correlation coefficient of expression values between two independent biological duplicates. Similarities in the expression profiles were analyzed by generating heat maps using R [22]. Correlation similarity matrix comparing different compounds, or SAR analogues was evaluated using both R and the Pearson product moment correlation coefficient.

## Results

### A feature reduced compendium of chemical-genetic profiles

**Figure 1:**
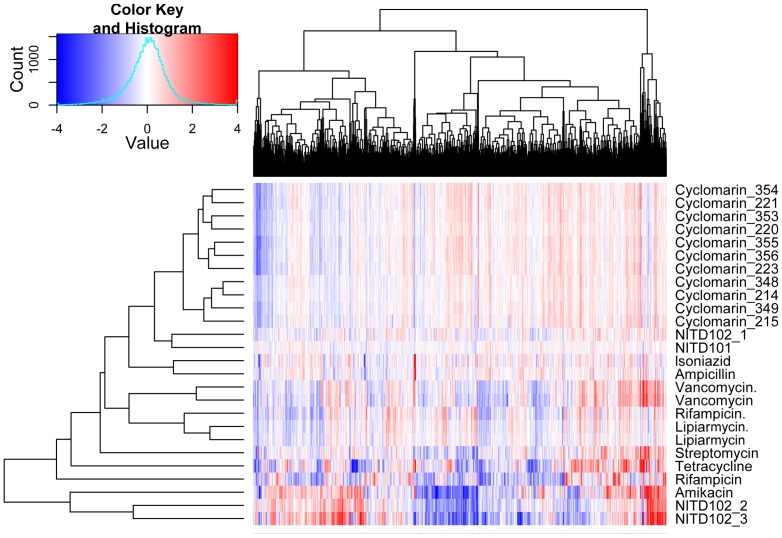
Cellular transcriptional responses of differentially-expressed genes clustered by inhibitor mechanism of action. A two-way clustering heat map of chemically perturbed expression profiles using the data from whole genome microarray is shown. Agglomerative clustering was performed on the 1860 genes differentially expressed in response to the given drug treatments. Profiles were clustered using Euclidean distance in the similarity matrix. The individual genes are represented on the x- axis and the different drug treatments are indicated on the y-axis (columns). Red, up-regulation; Blue, down-regulation; White, no change relative to the control. All expression values were transformed to log base 2.

We established the transcriptional response of *M. tuberculosis* to 12 different inhibitors. Included in this collection were 5 synthetic compounds and natural products derived from phenotypic screens, the rest were FDA approved drugs with anti-tubercular activity. The list of inhibitors used for perturbing bacteria and MoA diagnosis are included in Table 1. Microarray results showed differential expression for 1860 genes out of 4734 genes that were used in the array. (GEO accession # GSE46212 and GPL1343). The hierarchical heat map of bacterial transcriptional response of 1860 genes to the list of antibacterials showed that groups of drugs clustered separately based on their known mechanism of action (Fig. 1). Thus, protein synthesis inhibitors, transcriptional inhibitors, cell wall synthesis inhibitors and novel antibacterials fell into distinct groups. Boshoff and co-workers had previously described the application of transcriptional profiling using microarrays for MoA diagnosis of a natural product extract with no known mode of action [11]. However, the approach is not easily scalable and suitable for routine MoA diagnosis for hundreds of compounds from phenotypic screening. To exploit this property amenable for phenotypic screening, we limited the number of genes defining a condition to 90 using feature reduction.

**Table 1:** List of investigational and FDA approved anti-tubercular drugs and their mode of action.

Compounds	Mode of action	BCG MIC_50_ (µM)
NITD101	Unknown	-
NITD102	Unknown	-
NCE1a (AD841)	Unknown	0.02
NCE1b (AD1015)	Unknown	0.04
NCE1c (AD842)	Unknown	0.2
NCE2a (AD1158)	Unknown	0.38
NCE2b (AD1441)	Unknown	1.28
NCE2c (AD1259)	Unknown	2.06
NCE2d (AD1260)	Unknown	2.04
NCE2e (AD1534)	Unknown	7.2
NCE2f (AD1540)	Unknown	0.44
NCE3a (NITD_TB034)	Unknown	10
NCE3b (ATP425)	Unknown	1.6
NCE3c (ATP359)	Unknown	6
NCE3d (ATP435)	Unknown	2
NCE3e (ATP355)	Unknown	10
Cyclomarin- Series	Targets the ClpC1 subunit of the Caseinolytic Protease	
Liparmycin- Series	DNA-dependent RNA polymerase inhibitor	
PA-824	Possibly acts via generation of radicals having nonspecific toxic effects. Might possibly target enzymes in mycolic acid & protein biosynthesis.	0.44
Linezolid	Binds to the A site of the 50S subunit, and prevents the assembly of the ribosome initiation complex.	1.52
Ethambutol	Cell wall inhibitor, inhibits arabinosyl transferases involved in cell wall biosynthesis.	2.96
Isoniazid	Cell wall Inhibitor, Isoniazid inhibits *inhA*, a NADH-specific enoyl-ACP coA reductase involved in mycolic acid biosynthesis	0.28
Ethionamide	Cell wall inhibitor, disrupts of mycolic acid biosynthesis	2.04
Prothionamide	Cell wall inhibitor	0.9
Amikacin	**Aminoglycosides**: Inhibit translocation of the peptidyl-tRNA from the conserved A-site to the P-site in the 30S ribosomal Subunit.	0.38
Capreomycin		1
Streptomycin		0.16
Kanamycin		1.56
Clofazimine	Binds to guanine bases of bacterial DNA, causing steric hindrance in the template function of the DNA. Causes the accumulation of toxic lysophospholipids which inhibit bacterial growth.	0.26
Ampicillin	**Penicillin**: Beta lactam antibiotic. Penicillins bind to transpetidase enzymes (which are involved in the continuous remodelling of the peptidoglycan layer by cross linking peptides chains). [Note transpeptidase catalyses the terminal step of cell wall biosynthesis by cross linking the peptidoglycan]. By inhibiting this enzyme, penicillin prevents the formation of peptide bonds, weakens the cell wall, which subsequently cause lysis of the cell membrane.	
Moxifloxacin	**Quinolones**: Inhibit the bacterial ATP-dependent enzyme DNA gyrase and topoisomerase IV, preventing DNA unwinding during bacterial replication, transcription and bacterial DNA repair.	0.28
Ofloxacin		1.56
Levofloxacin		0.84
Gatifloxacin		0.2
Sparfloxacin		0.22
Valnemulin	**Pleuromutilin**: Binds to the peptidyl transferase component of the 50S subunit of ribosomes.	6.76
Vancomycin	**Glycopeptide**: Vancomycin binds with high affinity to the D-Ala-D-Ala C-terminus of the pentapeptide, thereby blocking the addition of late precursors by transglycosylation to the nascent peptidoglycan chain and preventing subsequent cross-linking by transpeptidation	
Rifampicin	Inhibits the essential *rpoB* gene product a subunit of DNA-dependent RNA polymerase activity	0.02
Rifabutin		0.02
Rifapentine		0.02
Thioridazine	A **phenothiazine** interfering with NADH dehydrogenase	14.08
TMC207	**Diarylquinoline** which inhibits the proton pump of ATP synthase.	0.32
Tetracycline	Bind to the 30S ribosomal subunit in the mRNA translation complex thus, inhibiting binding of aminoacyl-tRNA to the mRNA-ribosome complex.	
*p*-aminosalicylic acid (PAS)	Inhibitor of folic acid biosynthesis pathway	0.56

MIC_50_ listed for anti-tubercular investigational and SAR compounds, when available.

### Feature reduction and real-time qPCR for MoA deconvolution

**Figure 2:**
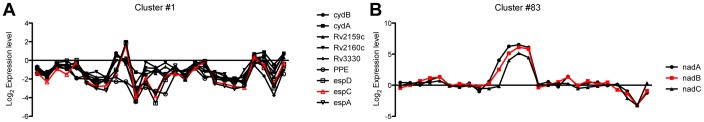
Transcriptional profile of gene clusters in response to antibiotics. X-axis denotes different drugs, and the y-axis denotes relative expression of the gene in response to the particular drug on the x-axis. Whole genome microarray data were used for this analysis. (A) A cluster replete with genes in respiratory metabolic pathway as well as subunits of the secretion system. The ESX section associated protein EspC was selected as the gene best representing the cluster transcription profile. (B) Genes of a related metabolic pathway cluster together in response to antibiotics. All three genes are from one metabolic pathway. NadB involved in quinolone biosynthesis served as the representative of the cluster.

Feature reduction attempts to identify a set of non-redundant genes that are useful for MoA classification. To reduce the features amenable to a robust 96 well format, a total of 90 genes from 90 clusters derived from agglomerative hierarchical clustering were selected. Briefly, the expression profiles of single genes was successfully joined to form nodes, which in turn were joined further until a total of ninety clusters were obtained. At the finest level, redundant gene representations across all perturbations clustered together with genes in each cluster showing similar expression profile. Two representative pattern of transcriptional profile of gene clusters in response to various antibiotics are shown in Fig. 2A & B. However despite the similar expression patterns, individual genes could be distinguished from each other with subtle differences in their expression profiles (Fig. 2A). To reduce the number of features a single gene was selected from each cluster that best represented the cluster expression profile by finding the gene most correlated with the mean expression profile of the cluster. The 90 genes selected with their known function are listed in Table S1.

**Figure 3:**
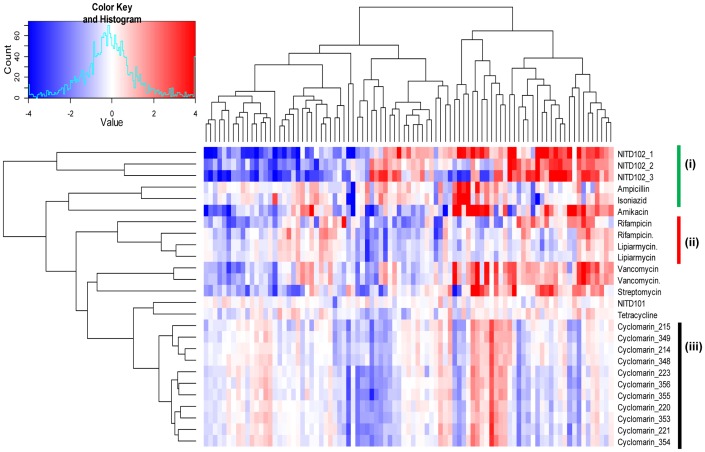
Transcriptional responses to drug treatment reveal that a single gene representing logically associated gene clusters is sufficient for MoA diagnosis in *Mycobacterium tuberculosis*. Groups of drugs clustered separately based on the known mechanism of action, while SAR series of compounds with novel MoA (cyclomarin) showed a fingerprint distinct from any of the compounds tested. Periods following drug names represent duplicates. X-axis represents the 90 genes that were chosen from the microarray as representative of 90 clusters, y-axis lists the different drug treatments. i, ii, and iii, represent 3 clusters namely, cell wall inhibitor, RNA polymerase inhibitor and Cyclomarin series, respectively.

To validate the functionality of the selected gene probes for MoA diagnosis, we analyzed bacterial transcriptional response to the chemical perturbation from microarrays. Hierarchical clustering analysis of the transcription reference compendium revealed that 90 genes are sufficient to define the MoA of hits derived from phenotypic screens (Fig. 3). We found that compounds with similar cellular effects showed similar transcription fingerprints and thereby cluster together on the vertical axis in revealing both anticipated and novel insights into their mode-of-action. In particular there were a couple of examples where the cluster analysis grouped inhibitors targeting the same pathway or target (Fig. 3). Individual clusters are annotated by roman numbers: (i) Cell wall inhibitors: Ampicillin (peptidoglycan) and Isoniazid (Mycolic acids); (ii) RNA polymerase inhibitors: Rifampicin and Lipiarmycin [23], and (iii) a series of ClpC1 caseinolytic proteasome inhibitors cyclomarin whose mode of action is novel [24]. The quality of the data was tested using Pearson rank correlation and Euclidean Distance, which verified that the expression fingerprint within each compound perturbation was tightly correlated (Fig. S1).

### MoA deconvolution of a natural product with anti-bacterial activity identified by chemical genetics

Given that, transcriptional profiling is applicable to any molecule that impairs bacterial growth, we evaluated the MoA of the natural product cyclomarin. To test whether chemical-genetic profiling may be particularly useful in driving the SAR for a molecule whose MoA was distinct from the compendium, we analyzed the transcriptional fingerprint of different derivatives of the natural product. All derivatives had a tight correlation, showing a similar fingerprint suggesting that they all had the same mode of action, and were probably targeting the same target (Fig. 3 & S1, Fig. 1). A detailed study on the elucidation of the MoA of the natural product cyclomarin is described elsewhere [24].

To validate the functional representation of the selected biomarker genes using qPCR, we evaluated the functional enrichment of genes. Table 2 summarizes the gene function enrichment of biomarker genes. Functional classes were defined according to Cole *et al* [25]. Gene function enrichment revealed that the dominant feature was a large percentage of virulence, detoxification and adaptation genes more than twice the expected number by chance. Genes from this functional class include; a heat shock protein *groES,* some conserved hypothetical proteins with PIN domains, a sub unit of the alkyl-hydrogen peroxide reductase *ahpC* involved in the oxidative stress response and genes in toxin-anti toxin operons. This demonstrates that the feature reduction can successfully enrich for genes involved in stress responses [26] as well as central pathways targeted by antibiotics [27].

**Table 2:** Pathway enrichment of functional classes of genes on PCR array.

Class ID	Functional Class	PCR array	Genome (H37Rv)
**0**	Virulence, detoxification & adaptation	10.59%	5.22%
**1**	Lipid metabolism	5.88%	5.84%
**2**	Information pathways	7.06%	5.17%
**3**	Cell wall and cell processes	20%	18.49%
**5**	Insertion sequences & phages	2.35%	3.62%
**6**	PPE/PE	3.53%	4.14%
**7**	Intermediary metabolism & respiration	15.29%	22.11%
**9**	Regulatory proteins	4.08%	4.78%
**10**	Conserved hypotheticals	15.29%	22.06%
**16**	Conserved hypotheticals with an orthologue in *M.bovis* (BCG)	8.24%	6.43%
**-**	Not annotated on H37Rv	7.06%	-

**Figure 4:**
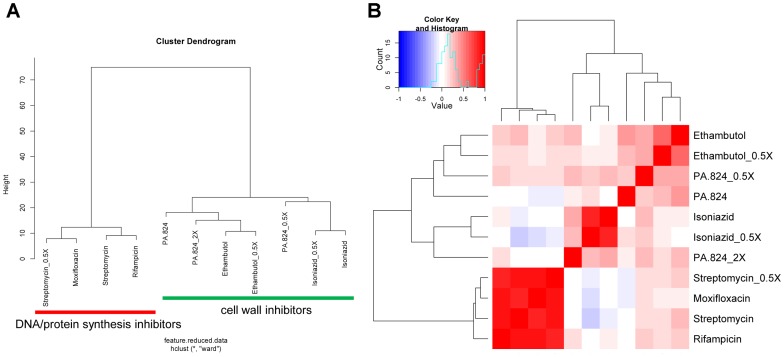
Transcriptional responses of *M. bovis* BCG to anti-tubercular drugs profiled using qPCR. A compendium of qPCR results can show differences in the MOA of anti-tubercular drugs. (A) Spatial grouping of drug expression profiles on a dendrogram after qPCR profiling using hierarchical clustering. From the list of drugs tested, two major clusters emerge cell wall inhibitors, and those that inhibit DNA/protein synthesis. (B) Similarity matrix of expression profiles for chemical inhibitors using qPCR profiling. The blue-red color scale shows the degree of correlation of drugs expression profiles ranging from −1 to 1 respectively. ×- depicts transcriptional responses at 2× or 0.5× the MIC_50_, while those that have no × designation were done 1× the MIC_50_.

One of the challenges of working with microarrays for MoA deconvolution is large sample volume, the need for multiple replicates, further compounded by the noise inherent in these systems. As a proof of concept to circumvent these properties, application of using oligos to PCR amplify the 90 selected genes as biomarkers to represent the whole genome was evaluated. A small collection of anti-tubercular drugs were analyzed by qPCR arrays using *M. bovis* BCG bacterial RNA. Analysis of the qPCR arrays showed that expression profiles from this platform were able to distinguish the MoA between anti-bacterial drugs (Fig. 4A & B). The minimal gene list to decipher the antibiotic mechanism of action on a PCR array has been summarized in Table S1.

### A medium throughput assay: Microfluidic platform

**Figure 5:**
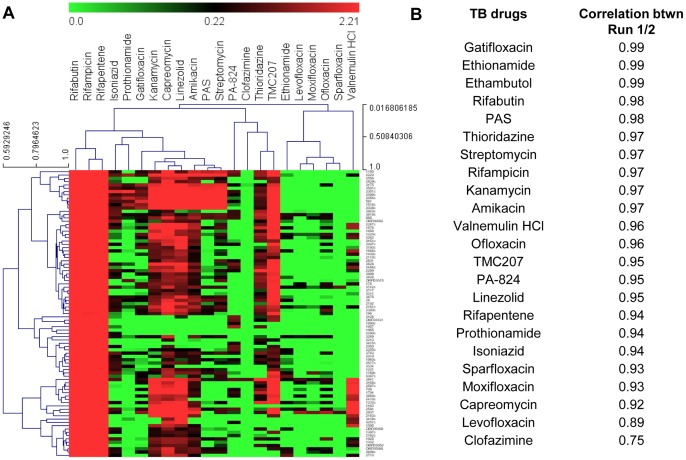
Hierarchical clustering and correlations of anti-tubercular drugs. Transcriptional responses data obtained from microfluidic experiment are used for the analysis. The detailed descriptions for genes represented in this figure are provided in Table S1. (A) Hierarchical clustering via average linkage of Pearson correlations for FDA approved drugs. The individual genes are represented in y-axis and the compound treatment is in the x-axis. (B) Pearson correlations for 23 anti-tubercular drugs. Correlations were calculated between 2 independent microfluidic experiments, following median normalization to account for plate effects.

Despite the excellent dynamic range, sensitivity and reproducibility of data using real time qPCR, this technique is a low throughput and expensive method to analyze a limited number of genes. Microfluidic digital PCR is able to generate higher throughput data technically identical in quality to the standard qPCR [19]. We explored the use of microfluidic chips to study the bacterial transcriptional response to a collection of anti-tubercular investigational and 23 FDA approved drugs (Table 1). As expected, drugs with similar mode of action, targeting the same cellular organelles clustered together (Fig. 5A). For example the fluoroquinolines clustered together, which inhibit the bacterial ATP-dependent enzyme DNA gyrase and topoisomerase IV, preventing DNA unwinding during bacterial replication, transcription and bacterial DNA repair. To validate the dynamic microfluidic PCR as a platform for routine gene expression analysis, the variation between and within measurement platforms for all genes under investigation using a subset of drugs was analyzed. The correlation between independent biological assays was as good as qPCR readouts (Fig. 5B).

### Driving SAR of a chemical genetics hits using microfluidic arrays

**Figure 6:**
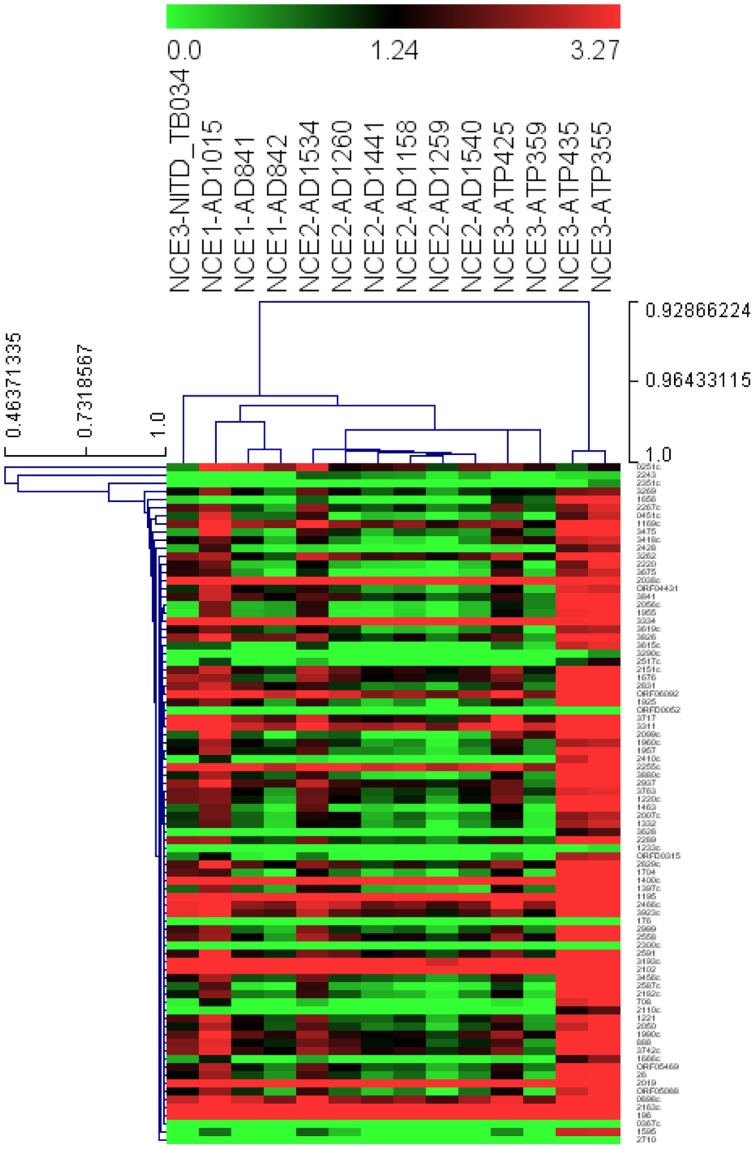
Transcriptional response of selected 90 genes to novel chemical entities. The heatmap was drawn using Pearson correlations through single linkage for both genes and compound treatments. The individual genes are represented in y-axis and the compound treatment is in the x-axis. Compounds from the same SAR library (designated as NCE1and NCE2) show a similar expression fingerprint, while NCE3 SAR compound series displays more variability in expression. The detailed descriptions for genes represented in this figure are provided in Table S1.

The principal advantage of a cell-based screen is that it represents an unbiased picture of both known and unknown cellular pathways that a molecule can modulate. The disadvantage of this empirical approach is that the target remains unknown. Hit to lead and lead optimization is generally more efficient when the target is known, thus target deconvolution is essential despite being time consuming and tedious. During SAR expansion, derivative molecules of the primary compound are made with a greater potency than the starting hit molecule (parent hit). It is imperative to monitor that these derivatives inhibit the same target or similar MoA, meaning that they have a similar transcription fingerprint as the parent hit molecule. In order to evaluate if SAR analyses can be improved for a compound with anti-tubercular activity, we evaluated the transcription fingerprint of a series of derivatives around particular pharmacophores. We hypothesized that a change in the transcription profile would indicate a change of target or MoA. To investigate this, a subset of an SAR expansion of NCE1, NCE2, and NCE3 were analyzed. SAR groups NCE1 and NCE2 showed a similar transcription fingerprint, indicating that they probably were still “on target” (Fig. 6 & S2). The NCE3 series displayed more variability in expression in our feature reduction microfluidic experiment; this is evident in both the heatmap and the correlation matrix (Figure 6& S2). All derivatives had anti-bacterial activity (Table 1).

## Discussion

There is an urgent need for new therapeutic approaches to antibiotic discovery as a result of the rapid emergence of multi-resistance to existing drugs, which is further exacerbated by the current gap in the development of new molecules. Phenotypic screening has re-emerged as the standard anti-bacterial drug discovery approach to avoid some of the attrition in target-based lead identification. When successful, it provides lead compounds with proven *in vivo* activity against the organism of interest [8], [9]. This approach has produced a large set of starting chemical points for anti-bacterial & anti-parasitic lead discovery [14], [28]–[32]. However, the cost of not having a biochemical assay for whole cell activity is that hit to lead and lead optimization becomes more challenging.

It is more difficult to establish consistent structure-activity relationships from phenotypic screens because whole-cell potency is a composite result of several compound properties, such as target affinity, cell permeability, efflux, and or intracellular modifications. Nonetheless, evidence supports the effectiveness of this approach in the identification of novel chemical classes with anti-bacterial or anti-malarial activity [23], [24], [28]–[32]. The key to success is a good chemical starting point and an adequate progression path to identify the potential to become a drug as quickly as possible.

The current study describes a more efficient tool for MoA deconvolution by transcriptional profiling of compounds derived from phenotypic screens using a microfluidic platform with integrated fluidic circuits. We show that the approach is indeed feasible from both a conceptual and practical point of view. A major goal was to identify a minimal set of genes that can be used as biomarkers for MoA deconvolution on an amenable miniaturized assay. Analysis of the whole genome bacterial transcriptional responses to these compounds helped in selecting a set of biomarker genes important in the general bacterial response to antibiotics, which was utilized in the development of a routine assay. Unsupervised grouping by hierarchical clustering revealed gene clusters whose expression profile for these inhibitors was consistent with previous studies on the MoA of these agents. More importantly, the clustering grouped together inhibitors with a similar mode of action.

A common theme, in all compound expression fingerprints was the remarkable over representation of the genes in bacterial defense, detoxification adaptation and virulence systems. Interestingly, a majority of the functionally characterized biomarkers appeared to be only indirectly related to the primary molecular targets of most of the inhibitors used. These were largely identified owing to their consistent expression pattern when exposed to antibiotic stress. Some of the biomarkers included heat shock proteins, toxin anti-toxin regulons, and detoxification systems against reactive oxygen species. These findings are in line with an increasingly more acknowledged mechanism of anti-bacterial action where the accumulation of broadly toxic intermediates and a suicidal derailing of central homeostatic system result in bacterial death [4], [7], [8], [27].

Hit to lead and lead optimization programs characteristically result in the generation of multiple compound series. Whilst it is important that cellular penetration, physiochemical profiles, and target inhibition are maintained or improved during SAR expansion, the gathering of this information is critical for good lead selection. Biomarker gene expression fingerprinting revealed that hit to lead and lead optimization could be guided in part by transcriptional profiling of the SAR series using the biomarker genes (Fig. 6). Derivatives of NCE1 and NCE2 showed similar fingerprints to the primary hit molecules, suggesting that they retained the same MoA. The tools described enable MoA diagnosis whilst prioritizing compounds from an SAR library of phenotypic screen derived leads. This will help in distinguishing the new series from other compound classes based on the transcriptional outcomes reflected in the gene expression by the chemical perturbations. Although the microfluidics, helped in tracking whether the novel compounds perturbed similar genes, it fails to give more details regarding the exact mode of action. Once the final compound has been identified, other techniques such as spontaneous mutant generation followed by whole genome sequencing and or affinity based target pull down studies need to be employed to identify the actual target.

The low number of biomarker genes required for MoA deconvolution and the establishment of a simple, robust and routine assay highlights the value of this technology in anti-infective drug discovery programs. As evidence to the potential application of this diagnostic platform, Barczak and co-workers recently underscored the application of drug RNA signatures for rapid clinical diagnosis of antibiotic susceptibilities in a broad range of bacterial pathogens [33]. The continued expansion of the drug transcriptome (collection of transcriptional fingerprints profiles generated by different chemical entities) using microfluidic arrays will generate a more composite resource for highly detailed functional cataloguing of compounds; such as grouping compounds that perturb similar sets of genes, as well as identifying sets of deletion mutants that show sensitivity to similar sets of compounds. Such tools can be used to augment phenotypic screens and are likely to lead to a wider application of MoA diagnostic assays in phenotypic screen campaigns. Although the present study presents evidence from anti-bacterial compounds, the vital components of this approach are not prokaryote specific. Analogous approaches can be applied in eukaryotes for other important medical indications such as the development of anti-malarial and anti-parasitic drugs.

## Supporting Information

Figure S1 A prediction correlation matrix using feature-reduced data to validate the classification of individual inhibitors. A heat map representation of gene expression similarity matrix of chemical inhibitors using selected features. The blue-red color scale shows the degree of correlation of drugs expression profiles ranging from −1 to 1 respectively. All expression values were transformed to log base 2. Periods following drug names represent duplicates. (TIF)

Figure S2 A correlation matrix based on Pearson correlations of compounds from the same SAR library. Note high levels of correlation as evident by the SAR clustering. NCE1 SAR compounds and NCE2 SAR compounds form tight clusters while NCE3 SAR compounds form two clusters. Red blocks indicate correlations greater than 0.98, blue blocks indicate correlations between 0.9–0.98, and white blocks are lower than 0.9. (TIF)

Table S1 Minimal number of biomarker genes for MoA deconvolution on a PCR array. Note the gene function were as depicted in <http://genolist.pasteur.fr/TubercuList/>. (DOCX)
