# Supplementary figures and images for: Exploring the Mode of Action of Bioactive Compounds by Microfluidic Transcriptional Profiling in Mycobacteria
Source: PLoS One. 2013 Jul 31;8(7):e69191. doi: 10.1371/journal.pone.0069191 (PMC3729944; doi:10.1371/journal.pone.0069191)

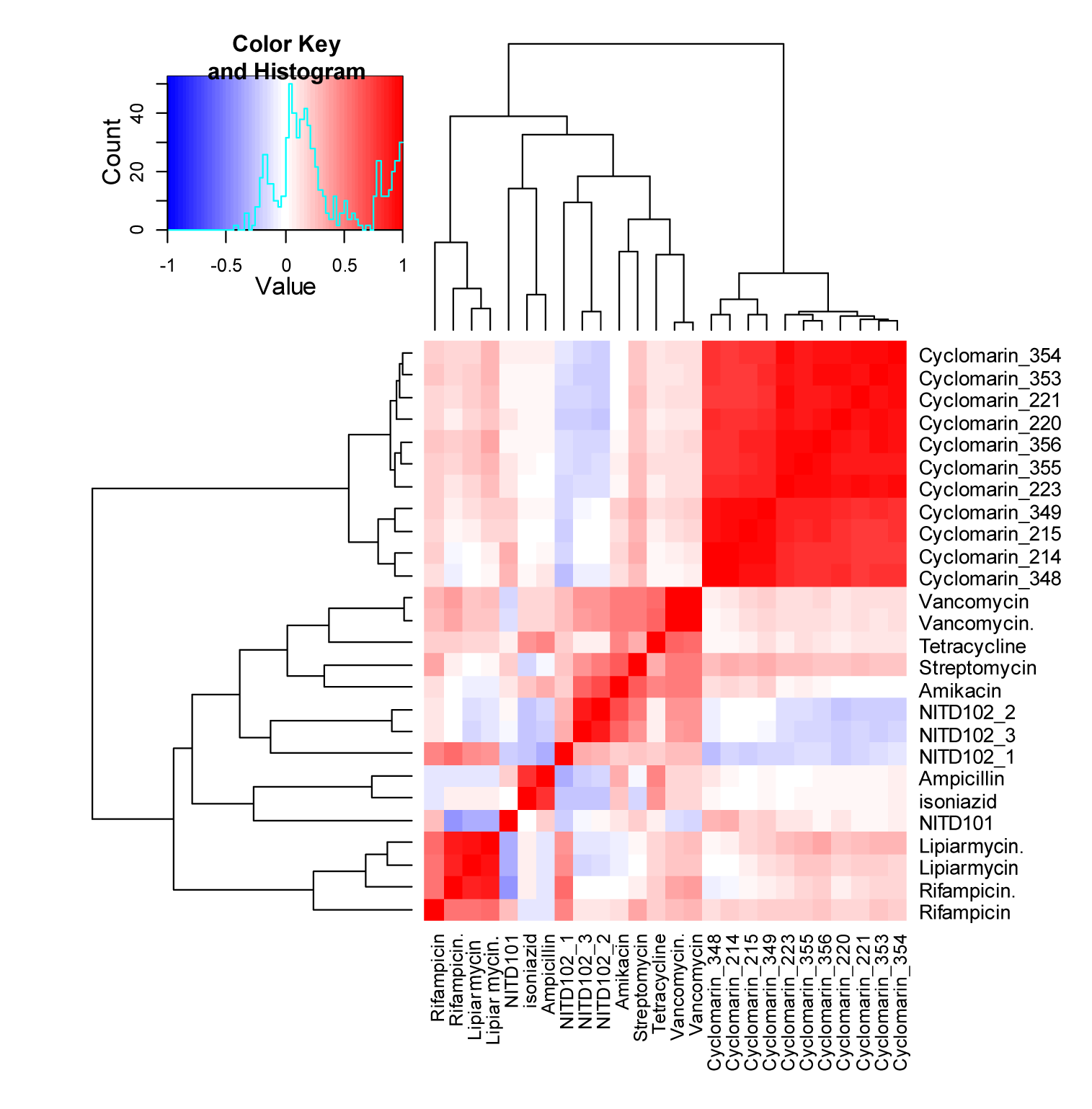

Supplement: Figure S1 — (TIF) [file pone.0069191.s001.tif]

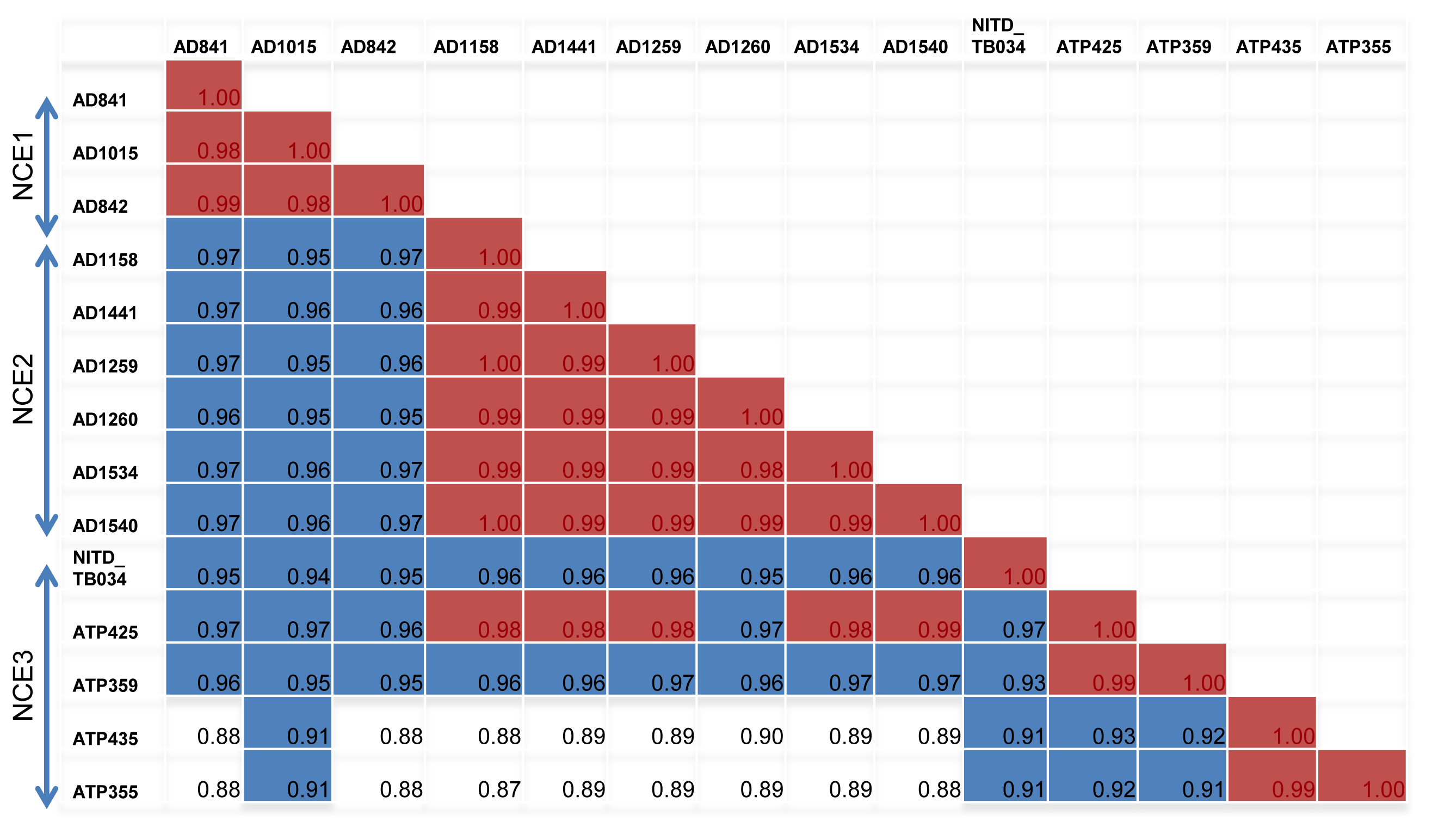

Supplement: Figure S2 — (TIF) [file pone.0069191.s002.tif]
