# Supplementary material for: Exploring the Mode of Action of Bioactive Compounds by Microfluidic Transcriptional Profiling in Mycobacteria
Source: PLoS One. 2013 Jul 31;8(7):e69191. doi: 10.1371/journal.pone.0069191 (PMC3729944; doi:10.1371/journal.pone.0069191)
Supplement: Table S1 — (DOCX) [file pone.0069191.s003.docx]

**Table S1**: Minimal number of biomarker genes for MoA deconvolution on a PCR array. Note the gene function were as depicted in <http://genolist.pasteur.fr/TubercuList/>

| **Gene** | ***M. tuberculosis* H37RV Accession Number** | **Gene function** |
| --- | --- | --- |
| Rv3615c | Rv3615c | Function unknown |
| *plcA* | Rv2351c | Hydrolyzes sphingomyelin in addition to phosphatidylcholine. Probable virulence factor implicated in the pathogenesis of mycobacterium tuberculosis at the level of intracellular survival, by the alteration of cell signaling events or by direct cytotoxicity [catalytic activity: a phosphatidylcholine + H_2_O = 1,2- diacylglycerol + choline phosphate]. |
| MT0036 | Not present | Hypothetical protein in *M. tuberculosis* CDC1551 |
| Rv3675 | Rv3675 | Unknown |
| *lpqH* | Rv3763 | Shown to inhibit gamma interferon regulated HLA-DR protein and mRNA expression in human macrophages |
| *ahpC* | Rv2428 | Involved in oxidative stress response. *lpdC*\|Rv0462, *dlat*\|Rv2215, *ahpD*\|Rv2429, and ahpc\|Rv2428 constitute an NADH-dependent peroxidase and peroxynitrite reductase that provides protection against oxidative stress. |
| PE13 | Rv1195 | PE family protein |
| PPE19 | Rv1361c | PPE family protein |
| *lprJ* | Rv1690 | Unknown |
| Rv1516c | Rv1516c | Unknown; involved in cellular metabolism. |
| Rv0581 | Rv0581 | Unknown |
| MT1083.2 | Not present | Hypothetical protein in *M. tuberculosis* CDC1551 |
| Rv3475 | Rv3475 | Involved in the transposition of the insertion sequence IS6110. |
| *sigE* | Rv1221 | The sigma factor is an initiation factor that promotes attachment of the RNA polymerase to specific initiation sites and then is released. Seems to be regulated by *sigH* (Rv3223c product). Seems to regulate the heat-shock response. |
| Rv3619c | Rv3619c | Unknown |
| Rv1960c | Rv1960c | Function unknown |
| Rv2558 | Rv2558 | Function unknown; thought to be involved in the persistence in the host. |
| Rv3269 | Rv3269 | Function unknown. May be involved in a chaperoning process. |
| Rv2466c | Rv2466c | Function unknown. Seems regulated by sigh (Rv3223c product). |
| MT2042.1 | Not present | Hypothetical protein in *M. tuberculosis* CDC1551 |
| Rv2255c | Rv2255c | Unknown |
| *fdxA* | Rv2007c | Involved in electron transfer. |
| *rplP* | Rv0708 | This protein binds directly to 23S ribosomal RNA and is located at the alpha site of the peptidyltransferase center. |
| Rv2410c | Rv2410c | Function unknown |
| PE_PGRS | Rv2591 | PE-PGRS family protein |
| Rv0888 | Rv0888 | Unknown |
| *Cdh* | Rv2289 | Involved in phospholipid biosynthesis [catalytic activity: CDP-diacylglycerol + H_2_O = CMP + phosphatidate]. |
| *glnA1* | Rv2220 | Involved in glutamine biosynthesis [catalytic activity: Atp + l-Glutamate + NH_3_ = ADP + glutamine + orthophosphate]. |
| *fadD23* | Rv3826 | Function unknown, but involved in lipid degradation. |
| Rv2267c | Rv2267c | Unknown |
| Rv1220c | Rv1220c | Function unknown; involved in cellular metabolism |
| Rv3717 | Rv3717 | Function unknown |
| *fabD* | Rv2243 | Catalyzes Malonyl-coA-ACP transacylase (mCAT) activity using holo-acpM as substrate for transacylation [catalytic activity: malonyl-coA + [acyl-carrier protein] = coA + malonyl-[acyl-carrier protein]]. |
| Rv2102 | Rv2102 | Unknown |
| *cycA* | Rv1704c | Permease that is involved in the transport across the cytoplasmic membrane of D-alanine, D-serine and glycine |
| Rv3262 | Rv3262 | Required for coenzyme F420 production: involved in the conversion of Fo into F420. |
| *argF* | Rv1656 | Involved in arginine biosynthesis [catalytic activity: carbamoyl phosphate + L-ornithine = phosphate +L-citrulline.] |
| *ftsQ* | Rv2151c | This protein may be involved in septum formation. |
| *groES* | Rv3418c | Binds to cpn60 in the presence of Mg-ATP and suppresses the ATPase activity of the latter. |
| Rv3880c | Rv3880c | Function unknown |
| *rplQ* | Rv3456c | Involved in translation mechanism. |
| *secD* | Rv2587c | Involved in protein export. Part of the prokaryotic protein translocation apparatus which comprise *secA, secB, secD, secE, secF, secG* and *secY*. |
| *Ppa* | Rv3628 | Involved in the function of cellular bioenergetics [catalytic activity: pyrophosphate + H_2_O = 2 orthophosphate]. |
| Rv3501c | Rv3501c | Unknown. Predicted to be involved in lipid catabolism. |
| *lipI* | Rv1400c | Function unknown, but possibly involved in lipid metabolism |
| Rv1666c | Rv1666c | Cytochromes P450 are a group of heme-thiolate monooxygenases. They oxidize a variety of structurally unrelated compounds, including steroids, fatty acids, and xenobiotics. |
| Rv2300c | Rv2300c | Function unknown |
| Rv3311 | Rv3311 | Function unknown |
| Rv1957 | Rv1957 | Unknown |
| Rv1397c | Rv1397c | Unknown |
| *fadD31* | Rv1925 | Function unknown, but involvement in lipid degradation. |
| *drrB* | Rv2937 | Probably involved in active transport of antibiotic and phthiocerol dimycocerosate (DIM) across the membrane (export). *drrA*\|Rv2934\|Mtcy19h9.04, *drrB* and *drrC*\|Rv2938\|Mtcy19h9.06 may act jointly to confer daunorubicin and doxorubicin resistance by an export mechanism. Probably responsible for the translocation of the substrate across the membrane and localization of dim into the cell wall. |
| Rv1463 | Rv1463 | Thought to be involved in active transport across the membrane. Responsible for energy coupling to the transport system. |
| Rv1676 | Rv1676 | Unknown |
| Rv0176 | Rv0176 | Unknown |
| Rv3193c | Rv3193c | Unknown |
| Rv1233c | Rv1233c | Function unknown |
| Rv2038c | Rv2038c | Thought to be involved in active transport of sugar across the membrane (import). Responsible for energy coupling to the transport system. |
| *prcB* | Rv2110c | Protein degradation |
| *pbpB* | Rv2163c | Involved in peptidoglycan biosynthesis |
| *Rv2659c* | Rv2659c | Sequence integration. Integrase is necessary for integration of a phage into the host genome by site-specific recombination. In conjunction with excisionase, integrase is also necessary for excision of the prophage from the host genome. |
| *echA16* | Rv2831 | Could possibly oxidize fatty acids using specific components [catalytic activity: (3s)-3-hydroxyacyl-coa = trans-2(or 3)-enoyl-coA +H_­2_O]. |
| Rv2182c | Rv2182c | Transfer of fatty acyl groups |
| MT3958 | Not present | Hypothetical protein in *M. tuberculosis* CDC1551 |
| *Rv3742c* | Rv3742c | Function unknown; probably involved in cellular metabolism. |
| *rnpA* | Rv3923c | catalyzes the removal of the 5'-leader sequence from pre-tRNA to produce the mature 5'terminus. It can also cleave other RNA substrates such as 4.5S RNA. The protein component plays an auxiliary but essential role *in vivo* by binding to the 5'-leader sequence and broadening the substrate specificity of the ribozyme [catalytic activity: endonucleolytic cleavage of RNA, removing 5'-extra-nucleotide from tRNA precursor]. |
| Rv2019 | Rv2019 | Unknown |
| *whiB7* | Rv3197A | Involved in transcriptional mechanism. |
| *lipX* | Rv1169c | PE family protein, possible lipase |
| Rv2517c | Rv2517c | Unknown |
| Rv1990c | Rv1990c | Involved in transcriptional mechanism. |
| *lat* | Rv3290c | Possibly involved in l-alpha-aminoadipic acid (l-aaa) biosynthesis. Catalyzes the transfer of the terminal amino group of l-lysine or l-ornithine to alpha-ketoglutarate [catalytic activity: l-lysine + 2-oxoglutarate = 2-aminoadipate 6-semialdehyde + l-glutamate]. |
| MT3754 | Not present | Hypothetical protein in *M. tuberculosis* CDC1551 |
| *lppY* | Rv2999 | Unknown |
| *bfrB* | Rv3841 | Involved in iron storage; ferritin is an intracellular molecule that stores iron in a soluble, nontoxic, readily available form. The functional molecule, which is composed of 24 chains, is roughly spherical and contains a central cavity in which the polymeric ferric iron core is deposited. |
| Rv2829c | Rv2829c | Unknown |
| PE21 | Rv2099c | PE family protein |
| Rv2050 | Rv2050 | Conserved hypothetical protein |
| Rv3334 | Rv3334 | Involved in a transcriptional mechanism. |
| Rv0898c | Rv0898c | Function unknown |
| Rv0196 | Rv0196 | Possibly involved in transcriptional mechanism. |
| Rv0367c | Rv0367c | Unknown |
| *nadB* | Rv1595 | Quinolinate biosynthesis. Catalyzes the oxidation of l-aspartate to iminoaspartate which is condensed with dihydroxyacetone phosphate to quinolinate under the action of quinolinate synthase a [catalytic activity: l-aspartate + H_2_O + O_2_ = oxaloacetate + NH_3_ + H_2_O_2_] |
| *sigB* | Rv2710 | The sigma factor is an initiation factor that promotes attachment of the rna polymerase to specific initiation sites and then is released. May control the regulons of stationary phase and general stress resistance. Seems to be regulated by sigh (Rv3223c product) and sige (Rv1221 product). Seems to regulate *katG*\|Rv1908c and the heat-shock response. |
| Rv0026 | Rv0026 | Function unknown |
| Rv1332 | Rv1332 | Involved in transcriptional mechanism. |
| *mmpS4* | Rv0451c | Unknown |
| *rpsN2* | Rv2056c | Involved in translation |
| Rv1955 | Rv1955 | Unknown |
| *hsp* | Rv0251c | Thought to be involved in the initiation step of translation at high temperature. Bound to 30s ribosomal subunit. Possibly a molecular chaperone. Seems to be regulated positively by *sigE*\|Rv1221 and negatively by *hspR*\|Rv0353. |
